# Hypoxemia after pneumothorax exsufflation: a case report

**DOI:** 10.11604/pamj.2017.28.240.11136

**Published:** 2017-11-16

**Authors:** William Ngatchou, Gildas-paulin Yondou Sandjo, Daniel Lemogoum, Pierre Youatou, Ahmed Sabry Ramadan, Regis Sontou, Maimouna Bol Alima, Alain Plumaker, Virginie Guimfacq, Pierre Mols, Michèle Ngassa

**Affiliations:** 1 Department of Emergency and Cardiac Surgery, St Pierre University Hospital, Université Libre de Bruxelles, Belgium; 2 Department of Emergency St Pierre University Hospital, Université Libre de Bruxelles, Belgium; 3 Department of Cardiology, Erasme University Hospital, Université Libre de Bruxelles, Belgium; 4 Department of Radiology, St Pierre University Hospital, Université Libre de Bruxelles, Belgium; 5 Department of Cardiac Surgery, St Luc University Hospital, Université Catholique de Louvain, Belgium; 6 Department of Cardiology, Ixelles University Hospital, Université Libre de Bruxelles, Belgium; 7 Department of Gastroenterology, Brugmann Hospital Brussels, Université Libre de Bruxelles, Belgium

**Keywords:** Pneumothorax, re-expansion, oedema, hypoxemia

## Abstract

We describe a 36-year-old patient who was admitted to the emergency ward for acute dyspnea due to a spontaneous pneumothorax. He was successfully drained but shortly after presented a severe hypoxemia due to pulmonary oedema secondary to pulmonary re-expansion. The physiopathology behind this complication is still unknown. We will try to describe this complication and its predictive factors.

## Introduction

Primitive spontaneous pneumothorax (PSP) is a gaseous effusion occurs in a patient in the absence of a specific triggering factor or without an underlying lung disease. Its incidence varies according to studies and gender: 18-28/100 000 cases/year for men and 1.2-6/100,000/year for women in the USA [1] and 7.4 to 18/100,000 per year for men and 1.2 to 6/100 000/year for women in an British studies [2]. Thoracocentis is the common and accepted therapy for very symptomatic patients, regardless of the extent of the pneumothorax [3]. Needle exsufflation is as effective as intercostal chest tube drainage in case of stable PSP [3]. Intercostal chest tube drainage is recommended if it fails [3]. Pulmonary re-expansion oedema (RPE) may occur after pneumothorax and/or pleural effusion drainage. The precise physiopathology has yet to be defined, but its prevalence and predictive factors have being described in recent studies [4, 5].

## Patient and observation

**Figure 1:**
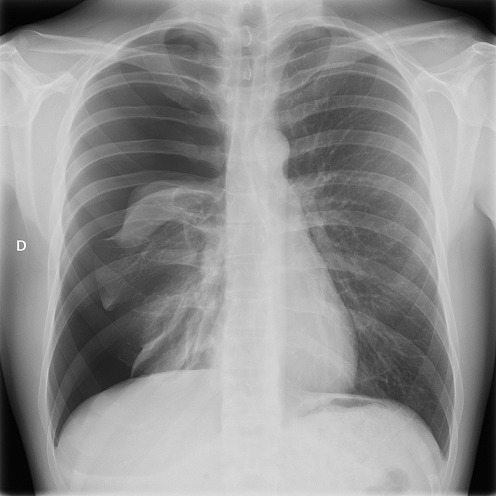
Thorax X-ray at the arrival

**Figure 2:**
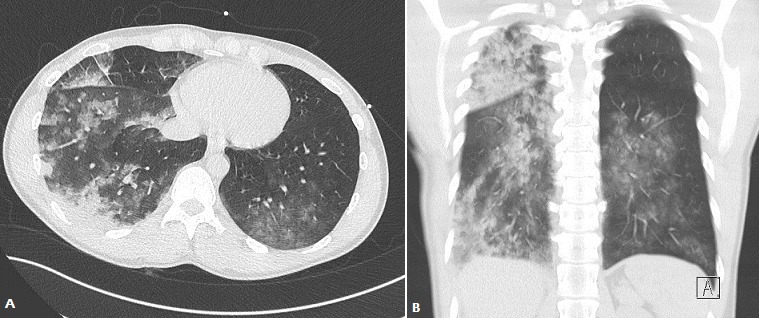
Axial (A) and coronal (B) non-contrast-enhanced–CT scan shows multiples images of alveolar opacity in the right lung and lesser in the contralateral lung

A-36-year old man was admitted to the emergency ward for dyspnea that started five days before without any notion of trauma. The patient had no medical history, but smoked 5-8 cigarettes a day since he was 18. His vital signs upon admission were: cardiac rate 70bpm, blood pressure 125/78, breathing rate 22/min and saturation 96% without oxygen. Pulmonary auscultation revealed hypoventilation on the right side, the blood sample was correct. The chest X-ray showed a complete right pneumothorax (Figure 1). After local anesthetic infiltration, a 6.3 Fr pigtail catheter (Cook Incorporated ^®^, Bloomington, USA) was placed through the anterior 2^nd^intercostals space and completed pulmonary re-expansion was obtained as confirmed by the chest X ray done 15 minutes later. One hour after the catheter insertion, the patient saturation dropped to 90-92% under 100% oxygen mask (arterial blood gas: ph: 7.4, PCO2: 40; PO2: 78, HCO3-: 25, Lactate 6). A ct scan were done and showed completed pulmonary re-expansion of the right lung. (Figure 2). The patient was admitted to ICU where he benefits from non-invasive ventilation. The drain was taken out after 72 hours and the patient left the hospital 6 days after his admission. He was seen 2 weeks later and his physical examination was strictly normal.

## Discussion

The aim of our case report is to remind us of a potentially severe complication of hemo or pneumothorax even if well drained. An incomplete drainage, a clogged drain or a misplaced drain are the first complications to exclude when a patient become hypoxic after chest tube insertion. The drainage system has to be verified, the patient re-examined and the thoracic imaging has to be obtained. In our case, clinical examination and radiography were satisfactory immediately after drainage. Two hours later, the patient became hypoxic, although pulmonary auscultation stayed symmetric and the drainage system was functional, inspiratory crackles could be heard, that's why a chest scan was obtained to exclude pulmonary oedema or residual pneumothorax not visible at the chest x ray. RPE is a syndrome characterized by the development of a pulmonary oedema most often after a rapid extraction air or liquid aspiration of a large volume (> 1.5L) from the pleural space [6]. The incidence of this syndrome varies between 0.9% and 29.8% in patients treated for spontaneous pneumothorax [4]. Risk factors included the delay before the drainage is instituted and the size of the pneumothorax [4, 5]. According to Moriaka, the delay between the onset of symptoms and drainage was 12.8 days ± 19.5 days in patients that had had RPE after a spontaneous pneumothorax against 8.8 ± 4.8 days in those without complication [4]. In Kim series [5], the mean delay was 5 ± 8.1 days corresponding to our case. The physiopathology behind RPE is still unknown. Several hypotheses have been proposed, including the lesion of the pulmonary blood vessels by rapid re-expansion of the lung tissue resulting in increased permeability [7], the endothelial vascular injury by increased oxygen free radicals and anoxic stress factors during the rapid re-expansion of the ischemic lung [8] and finally the capillary liquid passage into the interstitial tissues, due to the combination of re-perfusion pressure (hydrostatic pressure) in parallel with a low interstitial perivascular pressure [9]. Although these different mechanisms may each explain the formation of RPE, they still have to be validating by a clinical trial. Faced with a suggestive clinical picture, diagnosis should be confirmed on the basis of imaging after the elimination of differential diagnoses. Classically symptoms appear within the 24 hours following drainage, and can manifest as a persistent cough, dyspnea, chest pain, cyanosis, tachycardia, hypotension, nausea and vomiting [10, 11]. In our patient the very evocative clinical occurred about two hours after the establishment of the chest tube. Imaging although not formal is also evocative. In this current case, other diagnoses could be evocated such as an intra-alveolar hemorrhage secondary to a barotraumas due to an intrathoracic depression after important inspiratory efforts [12], or pulmonary contusion during chest tube insertion, or cardiogenic pulmonary oedema. But considering our patient's clinical history, these events were less likely. Treatment of RPE is essentially supportive consisting of a patient's hemodynamic stabilization, adequate oxygenation obtained by invasive or non invasive methods [10]. Patient's filling with plasma expanders (crystalloid and/or Colloids) and the use of amines may be necessary in case of hypoxemia and/or volume depletion generated by an intra-pulmonary shunt [10]. Positioning the patient in the lateral position on the affected side can reduce the shunt and improve oxygenation [13]. In our case, 3 days under non invasive therapy enabled us to have a good outcome. The prognosis of this disease is generally favorable [10-13].

## Conclusion

Pulmonary re-expansion oedema is a rare complication, but potentially dangerous that all surgeon or emergency physician should know. Its mechanism is not well understood and its treatment is generally supportive.

## Competing interests

The authors declare no competing interests.
